# Rescue of fragile X syndrome phenotypes in *Fmr1* KO mice by a BKCa channel opener molecule

**DOI:** 10.1186/s13023-014-0124-6

**Published:** 2014-08-01

**Authors:** Betty Hébert, Susanna Pietropaolo, Sandra Même, Béatrice Laudier, Anthony Laugeray, Nicolas Doisne, Angélique Quartier, Sandrine Lefeuvre, Laurence Got, Dominique Cahard, Frédéric Laumonnier, Wim E Crusio, Jacques Pichon, Arnaud Menuet, Olivier Perche, Sylvain Briault

**Affiliations:** 1 UMR7355, CNRS, Orléans, France; 2 Experimental and Molecular Immunology and Neurogenetics, University of Orléans, 3b rue de la Férollerie, Orléans, 45071, Cedex 2, France; 3 The Aquitaine Institute for Cognitive and Integrative Neuroscience, UMR 5287, CNRS, Talence, France; 4 The Aquitaine Institute for Cognitive and Integrative Neuroscience, University of Bordeaux, Avenue des Facultés, Talence, 33405, France; 5 Centre de Biophysique Moléculaire, UPR4301, CNRS, University of Orléans, Rue Charles Sadron, Orléans, 45071, Cedex, France; 6 Genetic department, Regional Hospital, 14 Avenue de l’Hôpital, Orléans, 45100, France; 7 UMR CNRS 6014 C.O.B.R.A., INSA of Rouen, 1 rue Tesnière, Mont Saint Aignan, 76821, France; 8 INSERM, U930, Tours 37032, France; 9 UMR Imagerie et cerveau, François-Rabelais University, Tours 37000, France

**Keywords:** Fragile X Syndrome, BMS-204352, BKCa channel, Sociability, Cognition, Anxiety

## Abstract

**Background:**

Fragile X Syndrome (FXS) is the most common form of inherited intellectual disability and is also associated with autism spectrum disorders. Previous studies implicated BKCa channels in the neuropathogenesis of FXS, but the main question was whether pharmacological BKCa stimulation would be able to rescue FXS neurobehavioral phenotypes.

**Methods and results:**

We used a selective BKCa channel opener molecule (BMS-204352) to address this issue in *Fmr1* KO mice, modeling the FXS pathophysiology. *In vitro*, acute BMS-204352 treatment (10 μM) restored the abnormal dendritic spine phenotype. *In vivo,* a single injection of BMS-204352 (2 mg/kg) rescued the hippocampal glutamate homeostasis and the behavioral phenotype. Indeed, disturbances in social recognition and interaction, non-social anxiety, and spatial memory were corrected by BMS-204352 in *Fmr1* KO mice.

**Conclusion:**

These results demonstrate that the BKCa channel is a new therapeutic target for FXS. We show that BMS-204352 rescues a broad spectrum of behavioral impairments (social, emotional and cognitive) in an animal model of FXS. This pharmacological molecule might open new ways for FXS therapy.

## Background

Fragile X Syndrome (FXS) is the most common cause of inherited mental deficiency and is associated with autistic features [1]. FXS is caused by a CGG triplet expansion in the *FMR1* gene resulting in the absence of its coding protein, Fragile X Mental Retardation Protein (FMRP). This mRNA-binding protein regulates both localization and translation of specific mRNAs in synaptic regions [2], but also controls synaptic membrane proteins activity through a translation-independent pathway [3]. As a consequence of this synaptic disturbance, a preponderance of long, thin and ’tortuous” dendritic spines in cortex is observed in FXS patients brain [4]. *Fmr1* knock-out (KO) mouse [5], a murine model of human FXS, presents both dendritic spines maturation abnormalities [6] and many behavioral characteristics similar to human FXS, including altered social interaction, occurrence of repetitive behaviors, hyperactivity and cognitive dysfunction [7],[8].

In the last decade, a better understanding of the FXS pathophysiology allowed to develop chemical therapeutics targeting specific synaptic components. Numerous studies have revealed that in the absence of FMRP, signaling through group 1 metabotropic glutamate receptors (mGluR 1/5) is disturbed. This dysfunction might underlie either the observed exaggerated synaptic long-term-depression or the insensibility of mGluR 1/5-mediated neuronal/synaptic protein synthesis stimulation [9]. Based on “other active mGluR 1/5 functions”, various selective molecules were tested *in vitro* on primary neuron cell cultures and *in vivo* on several behavioral defects in both *Fmr1* KO mice and FXS patients [10]-[12]. Although promising results were obtained, none of the tested therapeutic agents demonstrated a full effect on FXS behavioral, cognitive and molecular abnormalities. These reports prompt the interest of developing novel therapeutic targets.

Recent studies have demonstrated the implication of potassium channels in FXS pathology [13]. Among them, large-conductance Ca^2+^-activated K^+^ channels (BKCa channels, also known as BK or Maxi-K channels), activated by membrane depolarization and increased intracellular Ca^2+^ concentration, are of particular interest because of their control of Ca^2+^ concentration in neurons and regulation of neurotransmitter release such as glutamate [14],[15]. Functional BKCa channels are assembled as hetero-octamers of four *α*- subunits (KCNMA1 protein) and four auxiliary *β*-subunits, where the *β*-subunit is a tissue specific regulatory unit [16].

Several data provide convincing evidence that this channel is closely linked to behavioral and cognitive disorders. Our physical mapping of balanced chromosomal aberrations revealed a *KCNMA1* gene disruption in a subject with autism and intellectual deficiency. This gene haploinsufficiency induced a functional defect of BKCa channels that might contribute to neurological symptoms [17]. In addition, a mutation in the *CRBN* gene, an upstream regulator of BKCa channel, has been also associated with autosomal recessive non-syndromic mental retardation [18]. Behavior analysis obtained in mouse in which *Kcnma1* gene was deleted, showed a critical role for the BK channels in mechanisms underlying associative learning [19]. The hypothesis concerning BKCa involvement in FXS neuropathology was first proposed by Liao et al. [20]. In primary neuron cultures from *Fmr1* KO embryos, the authors observed a decreased expression of KCNMA1 protein. This defect suggested that some component of autistic and cognitive disorders seen in FXS might be, in part, due to BKCa channel activity abnormalities. Recently, Deng *et al.*[3] have demonstrated that BKCa current reduction in hippocampal slices results from an FMRP translation-independent effect. In hippocampal pyramidal neurons, and probably in cortical neurons, FMRP regulates action potential duration, neurotransmitter release and short-term plasticity through presynaptic BKCa channels. Based on all these data, we propose BKCa channel as a new pharmacological target in FXS therapy.

For this purpose, we used the fluoro-oxindole BMS-204352 ((3S)-(+)-(5-chloro-2-methoxyphenyl)-1,3-dihydro-3-fluoro-6-(trifluoromethyl)-2H-indol-2-one), as a selective BKCa channel opener [16],[17]. To investigate the therapeutic potential of this molecule, its effect was tested at first *in vitro* using a standardized primary neuron culture protocol focused on the *Fmr1* KO dendritic spines defects [6]. Based on promising *in vitro* results, we investigated its effect in *in vivo* conditions on *Fmr1* KO mice behavioral impairments as well as on metabolites homeostasis at hippocampus level [21]. In accordance with the main human clinical manifestations described in DSM-5 (Diagnostic and Statistical Manual of Mental Disorders), our behavior investigations concerned social/cognitive skills.

## Methods

For a full methodological description, see the supplementary content that accompanies the online edition of this article.

### Animals

All experiments were done with adult (3–5 months old) male *Fmr1* KO mice and their wild-type (WT) littermates of C57BL/6 J background [5]. *Fmr1* mice were obtained from the colony of the Aquitaine Institute for Cognitive and Integrative Neuroscience in Bordeaux (France). Breeding details were described in Additional file 1: supplementary methods. The present experimental protocol received full review and approval by the regional animal care and use committee (CREEA) prior to conducting the experiments. All possible efforts were made to reduce the number of animals studied and to avoid their suffering.

### Neuronal cell cultures and BMS-204352 treatment

Primary neuron cultures were prepared from male WT and *Fmr1* KO mice at embryonic day 15 (E15) as previously described [22]. Neurons were DiI-labeled and dendritic spine maturation were analysed using a protocol adapted from [6]. Experiments and analysis were done as blind to the genotype.

### Behavioral assays

The experiments were conducted in two laboratories: The Experimental and Molecular Immunology and Neurogenetics laboratory in Orléans, France (Laboratory A), and The Aquitaine Institute for Cognitive and Integrative Neuroscience in Bordeaux, France (Laboratory B). Mice were bred and maintained in the two laboratories following identical procedures.

In order to assess the social, cognitive and emotional components, we performed a direct social interaction test, the three-chamber test, the Y maze, and the elevated plus maze. The first two behavioral tests, i.e., the direct social interaction test and the three-chamber test for sociability and social recognition were conducted in parallel in both laboratories. This experimental strategy was adopted in order to assess the robustness and replicability of the results obtained. The last two tests, i.e., the Y maze for spontaneous alternation and the elevated plus maze were carried out in Laboratory B and Laboratory A, respectively. In order to minimize the number of animals studied, the same mice were used during the first three behavioral tests. Experiments and analysis were done as blind to the genotype.

All behavioral tests are described in Additional file 1: (Methods and Supplementary Results).

### Drug administration

The effective dose of BMS-204352 was chosen based on previously published data [23],[24]. BMS-204352 (2 mg/kg) diluted in the vehicle solution (DMSO 1/80; Tween 80 1/80; 0.9% NaCl) was administered by a 10 ml/kg single intraperitoneal (i.p) injection. Behavioral tests were performed at the maximal BMS-204352 brain concentration, i.e., 30 min after injection (Details appear in Additional file 1: Methods and Supplementary Results obtained by LC-MS/MS method, Additional file 1: Figure S1).

### Magnetic Resonance Spectroscopy (MRS)

MRS was realized as described previously with slight modifications [25] in order to quantify brain metabolites: glutamate, myo-inositol, N-Acetyl-Aspartate, taurine and lactate. Ten *Fmr1* KO and ten WT mice, which were not used in the behavioral tests, were included in the study. Metabolites concentrations are represented in arbitrary unit (AU), after normalization by Creatine/Phosphocreatine. Method is described in Additional file 1.

### Statistical analysis

For behavioral tests, data were analysed using three-way ANOVA with genotype, treatment, and laboratory as main factors for the three compartment and social interaction tests, and two-way ANOVA with genotype and treatment for the Y maze and elevated plus maze. Within-subject factors (e.g., contact area) were included when appropriate. Fisher PLSD’s *post-hoc* comparisons were used when a statistically significant main effect or interaction was detected (p < 0.05). For *in vitro* analyses, data were analysed using two-way ANOVA with genotype and treatment as main effects followed by Fisher PLSD’s *post-hoc* comparisons. All statistical analyses were done using Statistica 8 (StatSoft). For MRS, metabolites concentrations obtained in WT or *Fmr1* KO mice were compared using t-test or Mann–Whitney tests when non-parametric analysis was required. Treatment efficiency was evaluated by Wilcoxon tests.

## Results

### BMS-204352 reverses dendritic spine abnormal phenotype

**Figure 1:**
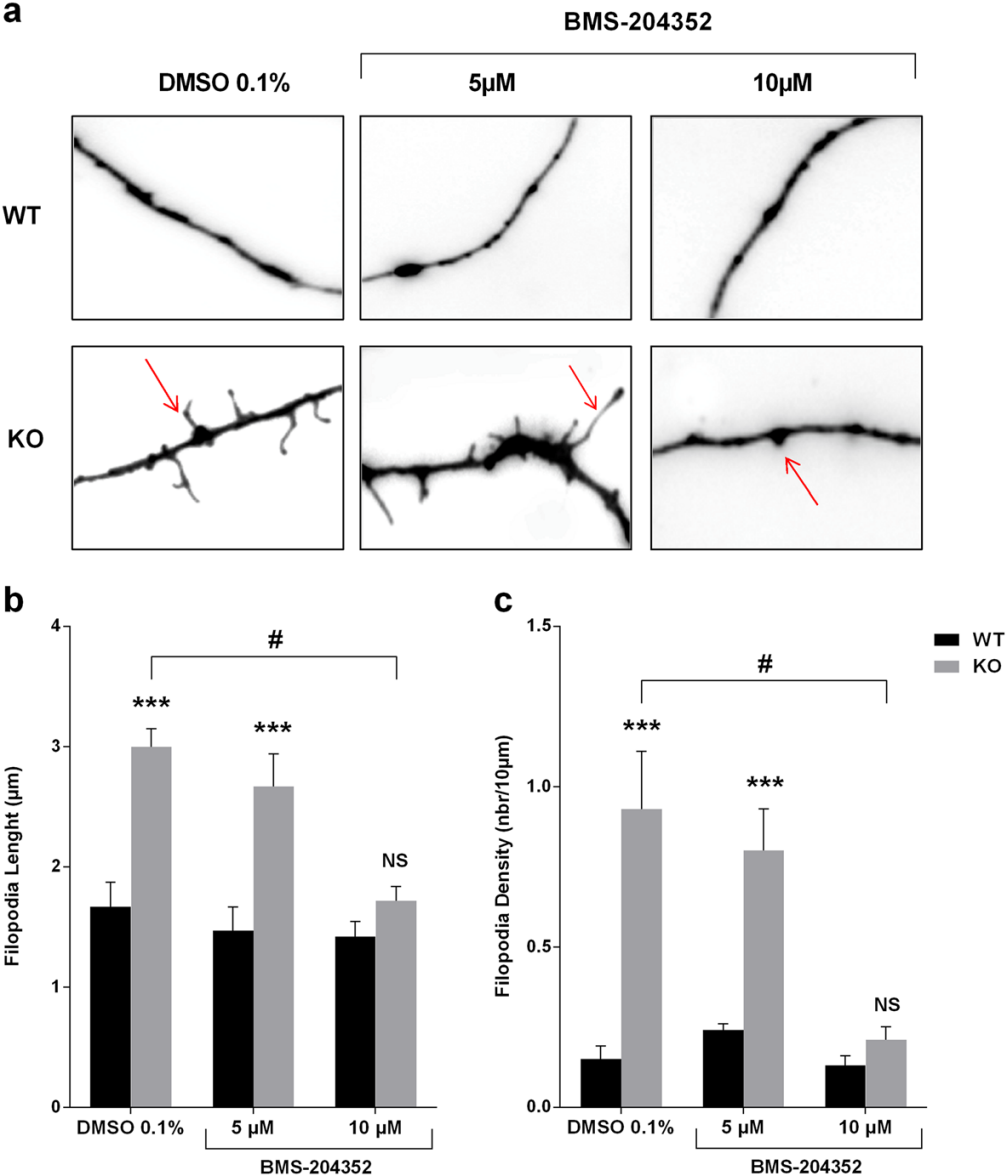
**BMS-204352 at 10 μM rescues dendrite spines maturation in Fmr1 KO neurons cultures. (a)** Representative pictures of neuron dendrites in different culture conditions with BMS-204352 (5 or 10 μM) or with only its vehicle (DMSO 0.1%). Red arrows indicate filopodia. Scale bar = 10 μm **(b)** Filopodia length (μm) and **(c)** density (nbr/10 μm) were investigated in each condition. A two-way ANOVA revealed that vehicle-treated *Fmr1* KO neurons showed a significantly higher filopodia length and density compared to vehicle-treated WT neurons. Acute treatment (4 hrs) with BMS-204352 10 μM corrected the *Fmr1* KO dendritic spine phenotype, whereas, BMS-204352 5 μM had no significant effect on *Fmr1* KO neurons. NS, not significant; ***p < 0.001 for genotype comparison; #p < 0.05 for treatment comparison; *n* = 60 neurons (from 10 mice) in all groups. Data represent mean ± s.e.m.

Increased dendritic spine density and length was reported in *post-mortem* analysis of FXS patients brain tissues [4] and observed in *Fmr1* KO mice *in vivo*[26] as well as *in vitro* on primary neuron cultures [6]. Vehicle-treated *Fmr1* KO neurons showed a significantly higher filopodia length (3.00 ± 0.15 μm *vs* 1.67 ± 0.2 μm, p < 0.0001) and density (0.93 ± 0.11 nbr/10 μm *vs* 0.15 ± 0.04 nbr/10 μm, p < 0.001) compared to vehicle-treated WT neurons (Figure 1). Acute treatment (4 hrs) with BMS-204352 10 μM corrected the *Fmr1* KO dendritic spine phenotype, whereas, BMS-204352 5 μM had no significant effect on *Fmr1* KO neurons. Indeed, BMS-204352 10 μM significantly reduced filopodia length (1.72 ± 0.12 μm, p < 0.01) and density (0.21 ± 0.04 nbr/10 μm, p < 0.01) of *Fmr1* KO neurons. In WT neurons, BMS-204352 (5 μM or 10 μM) acute treatment had no significant effect on the dendritic spine length or density.

### BMS-204352 reverses impaired social behavioral phenotype

**Figure 2:**
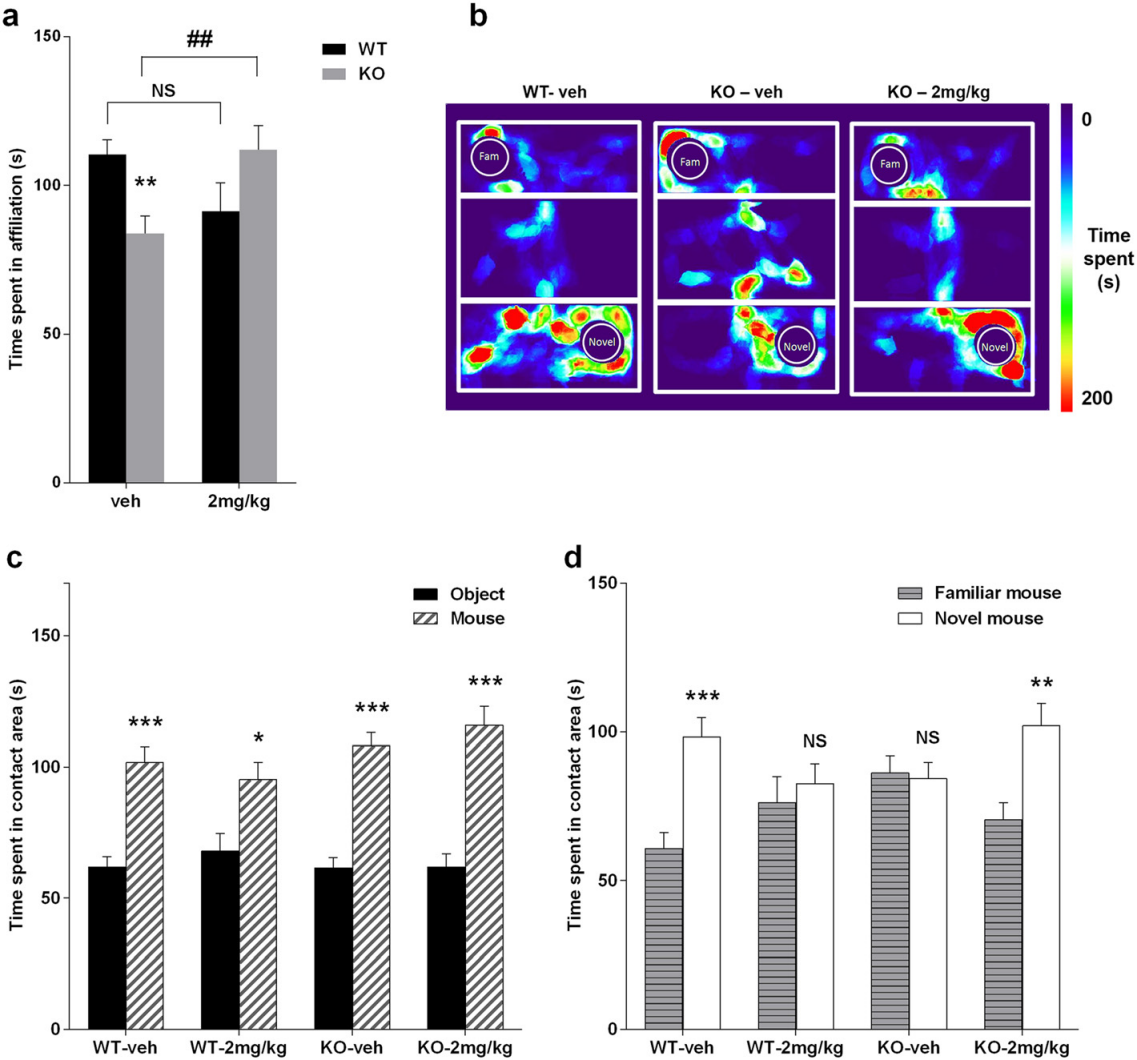
**BMS-204352 effects on Social behaviors.** Mice were administered with BMS-204352 2 mg/kg or vehicle (veh) and subjected to social behavioral tests 30 min after the injection. **(a)** Histograms represent time spent in affiliative behaviors obtained in two laboratories. A three-way ANOVA indicated a non-significant effect of laboratory, but a significant interaction between genotype and treatment. Vehicle-treated *Fmr1* KO mice spent less time in affiliative behavior compared to WT, but BMS-204352 injection restored a normal social investigation. (*n*: WT-veh = 27; WT-2 mg/kg = 16; KO-veh = 25; KO-2 mg/kg = 17). NS, not significant; **p < 0.05 compared to the corresponding WT; ^##^p < 0.05 compared the corresponding treated group. **(b)** Illustration of BMS-204352 effect on the three-chamber test by a pseudo-colored heat map representing time spent at each positions related to the social preference trial (fam: familiar mouse, novel: novel mouse). **(c)** Preference for a conspecific *versus* an object in the three-chamber test was measured by the time spent in the contact area when a stranger mouse and an object were accessible. Repeat-measures ANOVA indicated that vehicle-treated KO mice like WT shown a preference for the mouse *versus* the object, and that BMS-204352 treatment had no effect. **(d)** Preference for a novel *versus* a familiar mouse, was measured by the time spent in the contact area when a stranger and a familiar mouse were accessible. In vehicle-treated groups a preference for the novel mouse was only observed in WT. This preference was rescued by BMS-204352 treatment in *Fmr1* KO mice. (*n*: WT-veh = 34; WT-2 mg/kg = 19; KO-veh = 42; KO-2 mg/kg = 25). NS, not significant; *p < 0.05, **p < 0.01 and ***p < 0.001 (object *versus* mouse, familiar *versus* novel mouse). Data represent mean ± s.e.m.

Impairments in social interactions are core symptoms in FXS patients [1]. The assessment of *Fmr1* KO direct social interaction and BMS-204352 effect was conducted in two different laboratories (Laboratories A and B). A non-significant effect of laboratory [F(1,77) = 1.12, NS] and of its interactions with genotype [F(1,77) = 0.62, NS], treatment [F(1,77) = 0.39, NS] and both factors [F(1,77) < 1, NS] was observed. On the other side, a significant interaction of genotype and treatment was noticed [F(1,77) = 10.66, p < 0.01]: regardless the laboratory, vehicle-treated *Fmr1* KO showed significantly reduced social interactions with a stimulus female mouse compared to vehicle-treated WT animals [*post-hoc*, p < 0.01], and this reduced social investigation was corrected by BMS-204352 [*post-hoc*, p < 0.01], while a tendency to a negative effect of the treatment was observed in WT [*post-hoc*, p = 0.06] (Figure 2a). Indeed, a single BMS-204352 2 mg/kg injection in *Fmr1* KO mice was able to correct their deficits in social interactions, making them indistinguishable from WT mice. These results were confirmed in both laboratories, despite the obvious differences in environmental factors, i.e., experimenters (Additional file 1: Figure S2a-b).

To go further, we performed a second test in order to assay sociability, the three-chamber test. In the habituation phase (trial 1), all mice did not show a preference for any compartment or contact area in both laboratories (Additional file 1: Figure S2c-d). No difference between genotypes and treatments was observed for locomotor activity (Additional file 1: Figure S2g).

In trial 2, all mice preferentially explored the contact area containing the stimulus mouse compared to the one with the object, [main effect of contact area: F(1,112) = 54.11, p < 0.0001; Figure 2c], without difference between genotypes and treatments. A main effect of laboratories was observed [F(1,112) = 6.71, p < 0.05] since mice from Laboratory B spent more time in both contact areas, without interaction between genotype and treatments (Additional file 1: Figure S2e). These results confirmed the lack of deficits in sociability previously described in *Fmr1* KO mice in this test [8],[27]-[29]. Locomotor activity was similar in *Fmr1* KO and WT mice [main effect of genotype: F(1,112) = 2.25, NS ; Additional file 1: Figure S2h].

In trial 3, the social recognition trial, WT mice preferred to spent time in contact with the novel compared to the familiar mouse [main effect of contact area: F(1,112) = 8.81, p < 0.01], but this effect was modulated by both genotype and treatment [interaction genotype × treatment × contact area: F(1,112) = 10.02, p < 0.01]. Indeed, separate analyses in each group revealed that vehicle-treated WT mice showed a marked preference for social novelty, while this was absent in vehicle-treated *Fmr1* KO mice [interaction genotype x contact area: F(1,74) = 7.31, p < 0.01]. This deficit in social recognition was abolished by BMS-204352 treatment in *Fmr1* KO mice [interaction contact area x treatment: F(1,65) = 4.74, p < 0.05], as shown by the heat map illustration (Figure 2b), although a tendency to a negative effect was observed in WT mice [interaction contact area × treatment: F(1,51) = 3.27, p = 0.08; Figure 2d]. This negative effect in the WT mice was the consequence of an increase time spent in contact to familiar mouse *versus* a decrease time spent in contact to novel mouse (Figure 2d). All these effects were equivalently observed in both laboratories [all interactions with laboratory, NS; data not shown]. As in trial 2, a main effect of laboratory was observed [F(1,112) = 6.11, p < 0.05], without any consequences on the area preference or the differences between genotypes and treatments (Additional file 1: Figure S2f).

In this trial, locomotor activity was significantly higher in *Fmr1* KO mice compared to WT mice [main effect of genotype: F(1,112) = 5.76, p < 0.05], and this effect was not abolished by BMS-204352 treatment [interaction genotype x treatment: F(1,112) < 1, NS; Additional file 1: Figure S2i].

### BMS-204352 reverses impaired emotional behavioral phenotype

**Figure 3:**
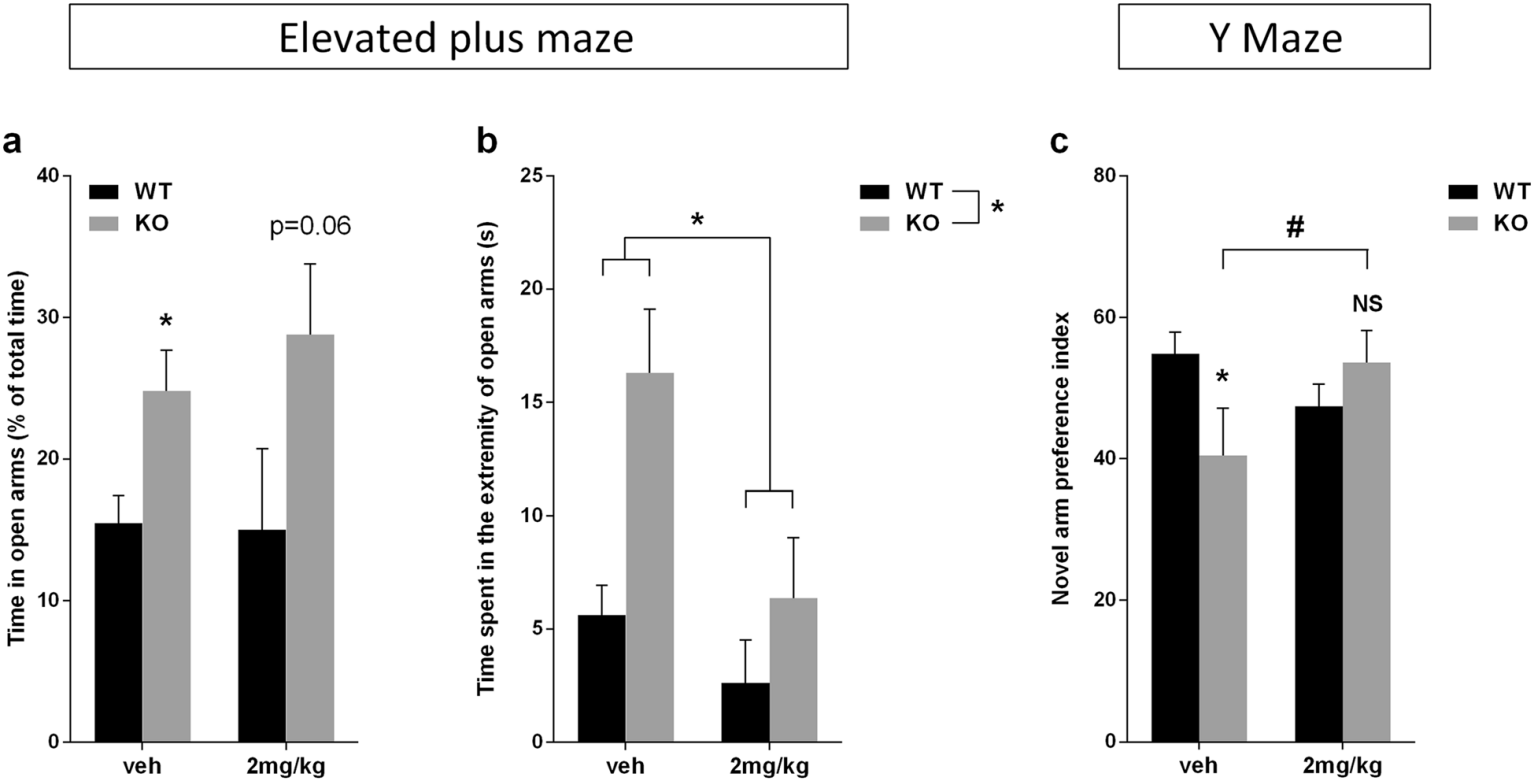
**BMS-204352 effects on Emotionality and Spatial memory. (a)** Percentage of time spent in open arms time in the elevated plus maze was calculated as the time in open arms/all arms x100. Analysis indicated a main effect of genotype, but no difference between genotypes and treatments was observed. **(b)** Time spent in the extremity of open arms was also evaluated and showed that *Fmr1* KO mice exhibited reduced non-social anxiety in comparison to WT mice. This was corrected by BMS-204352 treatment that increased anxiety in both genotypes. *p < 0.05 (*n*: WT-veh = 32; WT-2 mg/kg = 7; KO-veh = 32; KO-2 mg/kg = 10). **(c)** Preference index for a novel arm in the Y-maze was determined by the time spent in the novel arm/ time spent in all 3 arms. *Fmr1* KO mice presented spatial memory impairments through their incapacity to distinguish between novel and familiar arms. BMS-204352 treatment restored this cognitive defect to WT level. Significance was determined using two-way ANOVA with *post-hoc* PLSD test. NS, not significant; *p < 0.05, *vs.* WT; ^#^p < 0.05 *vs.* KO-veh (*n*: WT-veh = 7; WT-2 mg/kg = 6; KO-veh = 7; KO-2 mg/kg = 7). Data represent mean ± s.e.m.

The emotional component was evaluated by the elevated plus maze. As described in the literature [29],[30]*Fmr1* KO mice presented reduced non-social anxiety in this maze. Anxious mice naturally avoid open spaces and spend more time in closed arms, and less anxious mice spend more time exploring the open arms. The percentage of time spent in open arms is usually used as anxiety measure. The analysis demonstrated that *Fmr1* KO mice presented an elevated percentage compared to WT mice [F(1,77) = 8.51, p < 0.001; Figure 3a]. Also, they presented higher number of entries and time spent in open arms than WT mice [number of entries: F(1,77) = 4.04, p < 0.05; time spent: F(1,77) = 9.38, p < 0.01] (Additional file 1: Figure S3c and S3a, respectively), but the treatment had no effect [F(1,77) < 1, NS]. On the other hand, the time spent in closed arms did not differ between groups [F(1,77) < 1, NS; Additional file 1: Figure S3b]. Also, the amount of time spent in the extremities of the open arms has been established as a good index of anxiety [31]-[34]. *Fmr1* KO mice spent more time in the extremity of open arms [genotype effect: F(1,77) = 5.31, p < 0.05] and this was corrected by BMS-204352 that also increased non-social anxiety in WT mice [treatment effect: F(1,77) = 4.06, p < 0.05; Figure 3b].

For the distance moved, *Fmr1* KO mice were hyperactive compared to WT mice, and this genotype difference disappeared following BMS-204352 treatment [interaction genotype × treatment: F(1,77) = 8.08, p < 0.01]. Indeed, BMS-204352 significantly increased locomotion in WT mice, while tended to decrease it in *Fmr1* KO mice (Additional file 1: Figure S3d).

### BMS-204532 reverses impaired cognitive behavioral phenotype

The cognitive component was investigated through a hippocampus-dependent test, the Y maze for spontaneous alternation [35]. A two-trial memory task in a Y-maze, based on a free-choice exploration paradigm, has been previously developed to study recognition processes [36],[37]. *Fmr1* KO mice showed a deficit in spontaneous alternation and it was abolished by BMS-204352 treatment. The analysis of the percent novelty preference index demonstrated that spontaneous alternation was reduced in vehicle-treated *Fmr1* KO mice *versus* vehicle-treated WT mice, while returned to WT levels in BMS-treated *Fmr1* KO group, [interaction genotype x treatment: F(1,23) = 4.78, p < 0.05; *post-hoc*: p = 0.048; Figure 3c]. Moreover, the distance moved during the test did not differ between experimental groups: there was no effect of genotype or treatment [F(1,23) = 2.52, p = 0.13; F(1,23) = 1.17, p = 0.29 respectively] (Additional file 1: Figure S3e).

### BMS-204352 impacts brain metabolites level in vivo

**Figure 4:**
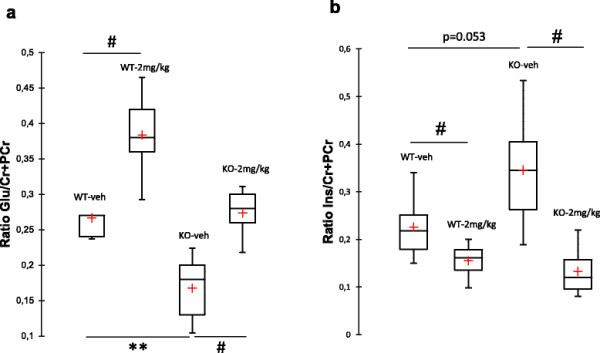
**BMS-204352 effects on glutamate and myo-inositol brain level.** Mice (3–5 months) were administered with BMS-204352 at 2 mg/kg or vehicle (veh) and subjected to MRS test 30 min after the injection. Box plots (shown as the minimum, 1st quartile, mean, median, 2nd quartile and maximum values) represent **(a)** glutamate and **(b)** myo-inositol concentrations in arbitrary unit (AU), after normalization by Creatine/Phosphocreatine, in vehicle or BMS-204352-treated-WT and *Fmr1* KO mice. Glutamate level was significantly decreased in *Fmr1* KO mice compared to WT, whereas myo-inositol concentration was increased. Furthermore, a single injection of BMS-204352 rescued a WT like metabolites concentration. Significance between WT and KO was determined using Mann–Whitney test and impact of treatment was determined by Wilcoxon test. NS, not significant; **p < 0.01 for genotype comparison; ^#^p < 0.05 for treatment comparison (*n*: WT-veh = 10; WT-2 mg/kg = 10; KO-veh = 10; KO-2 mg/kg = 10).

It has been suggested that glutamate signaling cascade in the absence of FRMP played a causal role in the behavioral phenotype of *Fmr1* KO mice [38]. *In vivo* effects of 2 mg/kg BMS-204352 treatment on glutamate concentration were studied by MRS experiments on the hippocampus structure. Vehicle-treated *Fmr1* KO mice presented a significant lower (p = 0.009) hippocampal glutamate concentration compared to vehicle-treated WT mice. Injection of 2 mg/kg BMS-204352 in *Fmr1* KO mice led to a significant (p = 0.043) increase of glutamate level (Figure 4a). Interestingly, this injection restored a WT glutamate level since no difference was observed between 2 mg/kg BMS-204352-treated *Fmr1* KO and vehicle-treated WT mice. An increased in glutamate was also observed (p = 0.043) after BMS-204352 injection in WT mice.

BMS-204352 single injection also restored the concentration of myo-inositol. Indeed, myo-inositol level was slightly higher (p = 0.053) with an important dispersion in vehicle-treated *Fmr1* KO mice compared to vehicle-treated WT littermates (Figure 4b). In *Fmr1* KO mice BMS-204352 induced a significant decrease (p = 0.002) in myo-inositol concentration, reaching a concentration similar to vehicle-treated WT level. A similar decrease (p = 0.038) was observed after BMS-204352 treatment in WT mice. Vehicle-treated *Fmr1* KO mice also presented significant deregulation of Taurine, N-Acetyl-Aspartate and Lactate metabolites (Additional file 1: Figure S4). In those mice, BMS-204352 (2 mg/kg) treatment had a significant effect on taurine and lactate concentration, restoring a WT level.

## Discussion

Currently, accumulated evidences highlight BKCa channel as a potential therapeutic target for FXS. Indeed, *in vitro* in the absence of FMRP, BKCa alpha subunit protein (KCNMA1) expression is decreased both in primary neuron cultures of *Fmr1* KO mice [20] and lymphoblastoid cells derived from FXS patients (Additional file 1: Figure S5). Moreover, Deng *et al.*[3] demonstrated that BKCa currents were decreased in CA3 pyramidal neuron of *Fmr1* KO mice. Dysregulation of neurotransmitter release and short-term plasticity, as a consequence of these defects, should in turn contributed to the behavior and cognitive deficits [3]. Therefore, we hypothesized that enhancing BKCa channel activity could rescue FXS neurobehavioral phenotypes. To this end, we chose to test the effects of fluoro-oxindole BMS-204352, a sensitive BKCa channel opener [17],[39], on the dendritic spines phenotype, behavioral impairments and the cellular hippocampal metabolism of the murine FXS model, the *Fmr1* KO mice.

At first, before conducting our *in vivo* experiments, we decided to evaluate BMS-204352 *in vitro* effects on dendritic spine morphology in primary neuron cell cultures from *Fmr1* KO embryos [4],[6],[40]-[42]. We showed that an acute treatment with BMS-204352 was able to restore the dendritic defects, typically observed in *Fmr1* neurons, to a regular level. This finding suggests that BKCa channel, which was previously defined as a determinant of presynaptic activity [15], should contribute to synaptogenesis. Hypothetically, these neuromorphological changes observed should directly link to local modulation of potassium flux, induced by an open conformational state of BK channel, as other potassium channels have been demonstrated to be closely associated with filopodia growth, dendritic development and/or neuronal differentiation [43],[44]. However, the link between local potassium ionic flux and the cellular pathway involved in dendritic maturation is worth being further explored.

The *in vitro* beneficial effects of several molecules on dendritic spine maturation of *Fmr1* KO mice were correlated with *in vivo* endophenotype observations [6],[45],[46]. BMS-204352 effects were investigated *in vivo* after intraperitoneal injection at 2 mg/kg [23],[24]. As previously described, adult *Fmr1* KO mice exhibited social behavioral impairments in a direct social interaction test as demonstrated by lower level of affiliative behaviors [27],[28],[47]. After injection of BMS-204352 2 mg/kg, affiliative behaviors in *Fmr1* KO mice were significantly increased to the WT level. To confirm and complete the results obtained in the direct social interaction test, we performed a three-chamber test, which showed social impairments in *Fmr1* KO mice demonstrated by their inability to distinguish a novel mouse from a familiar one, as previously described [27],[28],[47]. The observed effect in sociability components is very robust, as these tests were performed by different experimenter, in two different laboratories with two distinct cohorts of mice. Interestingly, during trial 2 (sociability trial), *Fmr1* KO mice and their WT littermates exhibited the same level of interest in the stimulus mouse compared to a non-social stimulus [27],[28],[47]. During this trial only olfactory and visual modalities come into play, and only the experimental mouse can initiate social contact. The reduced level of interactions of *Fmr1* KO mice during the direct social interaction test, is therefore most probably due to social anxiety induced by the direct contact with the stimulus mouse. In contrast, *Fmr1* KO mice exhibited reduced non-social anxiety compared to WT mice in the elevated plus maze [29]. Reduced non-social anxiety was rescued to normal levels after BMS-204352 treatment based on the time spent in the extremity of open arms. An acute treatment with BMS-204352 was therefore able to rescue the social deficits of *Fmr1* KO mice by increasing affiliative behaviors, decreasing social anxiety and enhancing social recognition. Our results are extended by spatial recognition test using Y-maze test. *Fmr1* KO mice presented spatial memory impairments through their incapacity to distinguish novel arm from familiar arms. This is in agreement with several studies which use different maze [6],[8],[48]. BMS-204352 treatment restored this cognitive impairment to WT level.

Therefore, we demonstrated that an acute BMS-204352 treatment at 2 mg/kg restored a normal phenotype in social, cognitive and emotional components by improving sociability, social and spatial recognition, and social/non-social anxiety.

Currently, distinct therapeutic targets are under investigations involving *Fmr1* KO mice. Many studies provide compelling evidence for several potential efficient pharmacological products. However, the comparison between those studies and ours was limited because they involved different mazes, molecules or strains. To our knowledge, our study is the first investigation to evaluate the efficiency of an acute treatment on the three main components of adult *Fmr1* knock-out mouse behavioral deficits: social, emotional and cognitive. Moreover, long-term study will provide additional data on phenotypic improvements already observed with a single-dose treatment. Indeed, positive behavioral effect due to BMS-20352 single injection were no longer observed 180 minutes after the injection (Additional file 1: Figure S6), likely due to the short BMS-204352 half-life (Additional file 1: Figure S1). Therefore, this long-term investigation will allow us to explore further cognitive traits such as learning and long-term memory, since Typlt *et al.* proposed a crucial role of the BKCa channels in learning [19].

As suggested before, in the absence of FMRP, numerous impairments of synaptic function were described with direct consequences on signaling cascades and cellular metabolism [3],[49],[50]. Moreover, presynaptic location of BKCa channels might provide a homeostatic mechanism for regulating synaptic transmission [3],[15]. Accordingly, we investigated BMS-204352 treatment effects on metabolic profile of *Fmr1* KO mice hippocampus with MRS method. The advantage of this non-invasive method was to obtain a metabolomic profile of a brain area in anaesthetized animals, unlike to other methods which required dissected tissues. By this MRS method, we showed that glutamate concentration was significantly reduced in our adult *Fmr1* KO mice compared to WT mice. A similar glutamate decrease was also described by ^1^H HR-MAS NMR spectroscopy on cortex of 12 days of age *Fmr1* KO FVB mice, suggesting that excitatory input appears compromised [50],[51]. Our result support the hypothesis previously published that glutamatergic function disturbance might contribute to FXS phenotypes in mice [3],[49],[50]. In our conditions, this glutamate reduction was rescued by a single injection of BMS-204352 at 2 mg/kg. Consistently with our behavioral experiments, BKCa channel opener should be a valuable tool to regulate glutamate neurotransmission and metabolism.

In addition, myo-inositol, mainly presents in astrocytes [52], was found deregulated in *Fmr1* KO mice. Myo-inositol concentration is altered in brain disorders such as Alzheimer’s disease and often, elevation of myo-inositol level reflects an astrocytic activation [53]. In our study, *Fmr1* KO mice also presented an elevated myo-inositol concentration suggesting an activation of astrocytes. This was consistent with glial fibrillary acidic protein (GFAP) and its mRNA up-expressions previously observed in several brain regions [51]. All of these data are consistent with an astrocyte involvement in synaptic defects observed in *Fmr1* KO mice [54]-[56]. Interestingly, BMS-204352 acute treatment decreased the myo-inositol concentration to WT level, however molecular and cellular pathways involved remained unclear. Based on these results, we suggest that BMS-204352 should be able to treat functional alteration of the tripartite synapse involved in the Fmr1 phenotype [55],[56].

## Conclusion

All these data reinforced evidences that BKCa pathway has to be explored as a new interesting therapeutic target for FXS patient. Therefore, our results suggested that BKCa channel opener molecule (BMS-204352) constitutes a promising potential medication for FXS patients correcting a broad spectrum of behavioral impairments (social, emotional and cognitive). BMS-204352 went up to phase III trial for the treatment of acute ischemic stroke but failed to show improvement against placebo. However during trials no organ toxicity or adverse effects were found [24]. This allows us to look forward with confidence to clinical trials involving few FXS patients.

## Competing interests

The authors declare that they have no competing interest.

## Authors’ contributions

BH, SP, JP, AM, OP and SB conceived and designed the experiments; BH, SP, AL and OP performed the behavioral experiments; SM and BH performed the SRMN; AQ, AM, ND and OP performed the *in vitro* experiments; DC produced deuterated molecule for kinetic assay; SL and LG performed molecule kinetic assay; BH, SP and OP analyzed the data; BH, AM and OP wrote the manuscript; SP, BL, ND, FL, WC, JP, AM, and SB coordinated and helped to draft the manuscript. All authors read and approved the final manuscript.

## Authors’ information

Betty Hebert and Susanna Pietropaolo are equal first contributors (co-first authors).

Olivier Perche and Sylvain Briault are equal contributors (co-final authors).

## Additional file

## Supplementary Material

Additional file 1: Additional Methods description and Results are presented in Additional file 1. Click here for file
