# Supplementary material for: Rescue of fragile X syndrome phenotypes in Fmr1 KO mice by a BKCa channel opener molecule
Source: Orphanet J Rare Dis. 2014 Aug 1;9:124. doi: 10.1186/s13023-014-0124-6 (PMC4237919; doi:10.1186/s13023-014-0124-6)
Supplement: Additional file 1: — Additional Methods description and Results are presented in Additional file 1. [file s13023-014-0124-6-S1.doc]

**Additional file 1**

**Supplementary Methods**

**Plasma and Brain sampling.**

Blood (cardiac puncture) and brain samples from mice were obtained prior to dosing and postdose (2mg/Kg, bolus) at 5, 30, 60, 90, 120, 180, 240 and 360 min (n=6 per time). Blood samples were drawn into Vacutainer® test tubes with Li-heparin. The samples were centrifuged at 2000 g for 10 minutes at 4 ºC, and plasma was separated and stored in Eppendorf tubes at -20º C until analysis. Each brain were carefully rinsed with saline solution and weighted individually. After homogenisation with 1mL of saline solution (NaCl 0.9%) for 400mg of brain, samples were frozen at -20º C until analysis.

**BMS assays: plasma and brain samples.**

BMS was quantified in plasma and brain samples using a sensitive and selective LC-MS/MS method. Briefly, to 50µL of plasma or brain samples were added the internal standard BMS –D3 (IS) and 100µL of acetonitrile. After centrifugation, 10µL of the supernatant was directly injected into the LC-MS/MS system equipped with an ion electrospray interface in negative mode. (5500 QTRAP, ABsciex, Saint Quentin en Yvelines, France) and an ultra high-performance liquid chromatography (Shimadzu, Saint Quentin en Yvelines, France). Data were acquired in multiple reaction monitoring including: m/z 358.9 → m/z 339 and m/z 358.9 → m/z 324 for BMS and m/z 360.9 → m/z 340.9 for the IS. The chromatographic separation was performed on a C18 BEH (50mm x 2.1mm; 1.7µm) column (Waters, Saint Quentin en Yvelines, France) maintained at 35°C. The mobile phase was composed of a mixture of solvent A (water, formic acid (99.9/0.1, v/v)) and solvent B (acetonitrile, formic acid (99.9/0.1, v/v)), delivered at a flow rate of 0.35 ml/min in the following gradient mode: 20% to 100% of B from 0 to 1min, followed by linear gradient to 100% of B from 1 to 2.8 min. The column was equilibrated from 2.9 to 4.5 min with 20% of B. The retention time of BMS was 2.8 min. Calibration curves were linear from 0.5 µg/ml (limit of quantification) to 50 ng/ml range. Within and between-day accuracy and precision at three levels: (0.5; 7; 30 ng/ml) were within acceptable limits (<15%).

**Animals**

All experiments were done with adult (3-5 months old) male *Fmr1* KO and their wild-type (WT) littermates of C57BL/6J background. *Fmr1* mice were obtained from the colony of the Aquitaine Institute for Cognitive and Integrative Neuroscience in Bordeaux (France). Breeding trios were formed by mating two heterozygous females with a WT male (purchased from Janvier, Le Genest St Isle). After 2 weeks the sire was removed, the females were single caged and left undisturbed until weaning of the pups. Mice were weaned at 21 days of age and group-housed with their same-sex littermates. On the same day, tail samples were collected for DNA extraction and subsequent PCR assessment of genotypes as previously described (Dutch-Belgian Fragile X Consortium 1994). All animals were housed in polycarbonate standard cages (26.7 x 20.7 x 14 cm in size; Tecniplast, Limonest, France), provided with sawdust bedding (SAFE, Augy, France), and a stainless steel wire lid. Food chow (SAFE, Augy, France) and water were provided *ad libitum*. Only male mice were used for the present studies; they were maintained in a male colony room under temperature (22°C) and humidity-controlled (55%) conditions with a 12:12 hr light–dark cycle (lights on at 7 a.m.). The present experimental protocol received full review and approval by the regional animal care and use committee (CLE CCO 2011-024) prior to conducting the experiments. All possible efforts were made to reduce the number of animals studied and to avoid their suffering.

**Neuronal cell cultures and BMS-204352 treatment**

Primary cultures of neurons were prepared from male WT and *Fmr1* KO mice at embryonic day 15 (E15) as previously described . Briefly, the entire brain was dissected out, and single cell suspensions from anterior brain were prepared by mechanical dissociation. Cells were washed in HBSS (10mM Hepes) and resuspended in culture medium (Neurobasal, Invitrogen, Fr) supplemented with 25mM glutamine (BioWest, Nuaille, Fr), B27 complement (Invitrogen, Carlsbad, CA), and 2mM penicillin and streptomycin (BioWest, Nuaille, Fr). Cells were seeded into poly-D-lysine-coated (0.5 mg/ml in borate buffer) coverslips in 24 well plates (60 000 cells/well) and incubated at 37°C in a moist chamber under 5% CO_2_. At 14 day *in vitro* (DIV) neurons were used for dendritic spine maturation study with or without BMS-204352 acute treatment (4 hr). BMS-204352 was diluted with DMSO 0.1% in supplemented Neurobasal to obtain 5 µM or 10µM in the culture well. After 4 hours of incubation medium was filled out, and neurons were prepared for DiI labeling.

**DiI labeling and dendritic spine maturation**

Neurons were labeled using a protocol adapted from Hering . Briefly, male WT and *Fmr1* KO neurons at 14 DIV were washed twice in HBSS, fixed in 4% paraformaldehyde for 15 min and incubated with Vybrant-DiI cell-labeling solution (1:200, Invitrogen) for 30 min at 37°C. Then cultures were washed in warmed PBS, incubated in PBS at 4°C for 24–48 hr to allow dye diffusion within membranes, mounted on glass slides with Fluoromount (Southernbiotech, Invitrogen), and then imaged using a Leica DM6000b microscope (Leica Microsystem, Nanterre, Fr). Filopodia spines length and density were measured using ImageJ Software (http://rsbweb.nih.gov/ij/). Six independent experiments were done sprayed over 6 months. Filopodia were counted in 10-12 neurons/wells with 6 wells/embryos. Filopodia were analyzed on 200 µm length of each neuron. The spine value was averaged in each independent experiment for the statistical test. The n value refers to the number of independent experiments analyzed.

**Behavior**

The experiments were conducted in two laboratories: The Experimental and Molecular Immunology and Neurogenetics laboratory in Orléans, France (Laboratory A), and The Aquitaine Institute for Cognitive and Integrative Neuroscience in Bordeaux, France (Laboratory B). Mice were bred and maintained in the two laboratories following identical procedures: the first two behavioral tests, i.e., the direct social interaction test and the three compartment test for sociability and social recognition were conducted in parallel in both laboratories. This experimental strategy was adopted in order to assess the robustness and replicability of the results obtained. The last two tests, i.e., the Y maze for spontaneous alternation and the elevated plus maze were carried out in Laboratory B and Laboratory A, respectively. In order to minimize the number of animals studied, the same mice were used during the first three behavioral tests.

In all behavioral assays, excluding the direct social interaction test, the mouse behavior was recorded by an analogic camera (SD5 - WV-CP500/G, Panasonic) and analyzed by a PC-based video tracking software (EthoVision XT, Noldus Technology, The Netherlands), monitoring also the distance moved and the velocity of the subjects.

**Drug administration**

The effective dose of BMS-204352 was chosen based on previously published data . BMS-204352 (2mg/kg) diluted in the vehicle solution (DMSO 1/80; Tween 80 1/80; 0.9% NaCl) was administered by a 10 ml/kg single intraperitoneal (i.p) injection. Behavioral tests were performed at the maximal BMS-204352 brain concentration, i.e., 30 min after injection (Details appear in Supplemental Experimental Procedures and Supplemental Data obtained by LC-MS/MS method, Figure S1).

**Direct social interaction test**

In the first behavioral test, we assessed mouse direct social interaction. As indicated above, this test was performed in two locations, i.e., in both our laboratories A and B. After the BMS-204352 or vehicle intraperitoneal injection, the subject (WT or *Fmr1* KO mice) was left undisturbed for 30 min into an individual polycarbonate cage (33.2 x 15 x 13 cm in size; Tecniplast, Limonest, Fr), provided with sawdust bedding, and a stainless steel wire lid. A stimulus female mouse (NMRI strain, 12 weeks) was then introduced in the cage for 5 min and mouse behavior was recorded by a numerical camera (Handycam, DCR-SR58E, Sony). Time spent in affiliative behaviors (sum of nose sniffing, anogenital sniffing, body sniffing and allo-grooming) was measured from the videos using the Observer XT software (version 10, Noldus Technology, The Netherlands) by a researcher who was blind to the genotype of test mice. Time spent in non-social behaviors, such as rearing, digging, and self-grooming was also computed.

**Three-chamber test for sociability and social recognition**

In order to evaluate mouse sociability and social recognition, we used the three-chamber test. The apparatus used in this study was similar to the one used by Nadler and was previously used to test *Fmr1* mice . The three-chamber apparatus was a non-transparent plexiglass box (25 x 50 cm) with two transparent partitions that make left, center, and right chambers (25 x 16.7 cm). Each partition had a square opening (5 x 5 cm) in the bottom center. A cylindrical wire cage (8 cm diameter; red pencil cup) was placed into the right and the left chambers. The test consisted of three phases. In the first 5 min session (habituation), the test mouse was placed in the center chamber, and was allowed to freely explore each chamber, with the wire cages empty. In the next 5 min session (sociability), an unknown juvenile male (DBA/2j strain), was placed in one of the two wire cages, while an inanimate object was introduced in the other wire cage. Testing in Laboratory B used a plastic grey cylinder as an object, while in Laboratory A a grey plastic mouse object was used. In the last 5 min session (social recognition), a second unknown juvenile male (DBA/2j strain) was placed in the wire cage where the object previously was. Thus, the test mouse would now have the choice between a mouse that was considered as familiar and a novel stranger mouse. The three-chamber apparatus and wire cups were cleaned with 70% ethanol then water and wiped with paper towels between each trial. Allocation of the stimuli was counterbalanced within experimental groups. Time spent in each chamber and time spent within a 3 cm radius proximal to each wire cage (contact area) was measured. Individual movement tracks were analyzed by EthoVision XT and modified by ImageJ software to generate heat maps.

**Elevated plus maze test**

In order to evaluate the anxiety phenotype of mice, we used the elevated plus maze test. This was a plus-shaped maze, 50 cm elevated above the floor, consisting of two closed arms surrounded by 21 cm high black walls and two open arms (6 x 35 cm). Each mouse was placed in the center (6 x 6 cm) of the maze facing one of the closed arms. During the 5-min test period, mouse behavior was recorded, and time spent in closed arms, center, open arms and extremity of arms was measured. In accordance to software parameters, a mouse was considered to be within an arm of the maze when all four paws were within the arm. The maze was cleaned with 70% ethanol then water and wiped with paper towels between each trial.

**Y Maze test**

We performed a Y maze test modified in order to assay spatial memory, an hippocampus-dependent spatial recognition component . A two-trial memory task in a Y-maze, based on a free-choice exploration paradigm, has been previously developed to study recognition processes . This included two phases: in the first 5 min session of acquisition, the animal was introduced at the end of the start arm and allowed to freely explore the two open arms of a Y maze, the third (novel) was blocked by a grey plastic door. The mouse was removed from the apparatus for a 10 min period (inter-trial interval), and left undisturbed in a waiting cage. Then the door was removed, and the mouse was reintroduced in the start arm and allowed to freely explore the entire maze for 2 min (retrieval). For both trials, the testing session started once the animal had left the start arm and first visited the center of the maze. The three arms of the Y maze were identical and spaced at 120° from each other. The Y maze was located in a room containing different extramaze cues. Each arm was 36.5 cm long and 7 cm wide, separated by a triangular center with 7-cm sides; all arms were enclosed by a wall of 12.5 cm height. Allocation of the start arm was counterbalanced within experimental groups. Time spent in the three arms was measured and used to obtain an index of percent novelty preference as follows: time spent in the novel arm/time spent in the three arms x 100. The maze was cleaned with 70% ethanol then water and wiped with paper towels between each trial.

**Magnetic Resonance Spectroscopy (MRS)**

MRS was realized as described previously with slight modifications . Ten *Fmr1* KO and ten WT mice, which were not used in the behavioral tests, were included in the study. Mice were placed on a home-built custom to immobilize their head. They were anesthetized during magnetic resonance experiment with 1.5% isoflurane and a mixture O_2_/N_2_O (1:1) with an output of 0.7L / min. Respiration motion was controlled during all the experiment using an air pillow. Mice body temperature was maintained constant with a warm water circulation. MR spectroscopy was performed on a 7T horizontal magnet dedicated to small animal imaging (70/16 USR Bruker Biospec, Wissembourg, Fr) and equipped with a 300mT/m gradient set. A Bruker 28 mm inner diameter bircage coil was used for both ^1^H transmission and reception. First of all scout images were performed to localize the voxel of interest using a RARE sequence with the following parameters: TR/TE = 5s / 56ms, rare factor = 8, FOV size = 2*2 cm, matrix size = 256*256, slice thickness = 1mm to display (78*78) µm in plane resolution for 5 min duration. Static B_0_ homogeneity was adjusted with first and second order shims in a (2.5*2.5*2.5) mm voxel centred in the hippocampus (bregma: -2.10 mm) with Bruker Fastmap procedure. The line width achieved for tissue water was less than 10 Hz. A PRESS sequence (Point Resolved Spectroscopy) was used to record localized ^1^H spectra in a cubic (2*2*2) mm voxel placed in fast map voxel with the following parameters (TR = 4s, TE = 16ms, 256 scan : 17min, 2048 points, bandwidth = 4000Hz) with water suppression using VAPOR (VAriable Pulse power and Optimized Relaxation delays) module and outer volume suppression. Eddy current compensation and static magnetic field drift correction were applied during spectra acquisition. ^1^H spectra were collected for WT and *Fmr1* KO pre and post treatment. Spectra were analyzed with JMRUI 3.0 software (http://www.mrui.uab.es/mrui/) working in time domain (baseline correction, phasing, zero filling.). AMARES module was used to quantify brain metabolites: glutamate, myo-inositol, N-Acetyl-Aspartate, taurine and lactate. Metabolites concentrations are represented in arbitrary unit (AU), after normalization by Creatine/Phosphocreatine, in box plots (the minimum, 1^st^ quartile, mean, median, 2^nd^ quartile and maximum).

**Proteins extractions and Expression (Western blot)**

Proteins extraction from murine neurons culture or human lymphoblastoïd cells were homogenized in ice-cold RIPA buffer (150mM NaCl, 50 mM TrisHCl, 5 mM EDTA, 0.1% SDS) supplemented with protease inhibitors (1% Protease inhibitor mixture, Fisher Scientific, Illkirch, France). Protein concentration was determined by Pierce^TM^ BCA pierce kit (ThermoScientific, France).

Protein extracts from murine neurons culture or human lymphoblastoïd cells (15µg) samples were separated in 12% SDS-PAGE, transferred to nitrocellulose membrane, incubated with 1:1000 diluted rabbit polyclonal anti-Kcnma1/anti-KCNMA1 antibody (Alomone Labs, France) or anti-FMRP antibody (ABCam, Paris, France) recognized by peroxydase-conjugated goat anti-rabbit antibodies. Load of equal amounts of protein was checked by 1:40000 diluted mouse anti-GAPDH antibody (ABCam, Paris, France). Quantification of immunoreactive bands was done using ImageJ (http://rsbweb.nih.gov/ij/). Blots were performed three times.

**References**

1. Ethell IM, Irie F, Kalo MS, Couchman JR, Pasquale EB, Yamaguchi Y: **EphB/syndecan-2 signaling in dendritic spine morphogenesis.** *Neuron* 2001, **31:**1001-1013.

2. Hering S: **Small-volume and rapid extracellular solution exchange around Xenopus oocytes during voltage-clamp recordings.** *Pflugers Arch* 1998, **436:**303-307.

3. Gribkoff VK, Starrett JE, Jr., Dworetzky SI: **Maxi-K potassium channels: form, function, and modulation of a class of endogenous regulators of intracellular calcium.** *Neuroscientist* 2001, **7:**166-177.

4. Jensen BS: **BMS-204352: A Potassium Channel Opener Developed for the Treatment of Stroke.** *CNS Drug Reviews* 2002, **8:**353-360.

5. Nadler JJ, Moy SS, Dold G, Trang D, Simmons N, Perez A, Young NB, Barbaro RP, Piven J, Magnuson TR, Crawley JN: **Automated apparatus for quantitation of social approach behaviors in mice.** *Genes Brain Behav* 2004, **3:**303-314.

6. Pietropaolo S, Guilleminot A, Martin B, D'Amato FR, Crusio WE: **Genetic-background modulation of core and variable autistic-like symptoms in Fmr1 knock-out mice.** *PLoS ONE* 2011, **6:**e17073.

7. Dellu F, Contarino A, Simon H, Koob G, Gold L: **Genetic differences in response to novelty and spatial memory using a two-trial recognition task in mice.** *Neurobiology of Learning and Memory* 2000, **73:**31-48.

8. Dellu F, Fauchey V, Moal ML, Simon H: **Extension of a new two-trial memory task in the rat: influence of environmental context on recognition processes.** *Neurobiology of learning and memory* 1997, **67:**112-120.

9. Dellu F, Mayo W, Cherkaoui J, Le Moal M, Simon H: **A two-trial memory task with automated recording: study in young and aged rats.** *Brain research* 1992, **588:**132-139.

10. Voineagu I, Wang X, Johnston P, Lowe JK, Tian Y, Horvath S, Mill J, Cantor RM, Blencowe BJ, Geschwind DH: **Transcriptomic analysis of autistic brain reveals convergent molecular pathology.** *Nature* 2011, **474:**380-384.

11. Gruetter R, Fusch C, Martin E, Boesch C: **Determination of saturation factors in 31P NMR spectra of the developing human brain.** *Magn Reson Med* 1993, **29:**7-11.

12. Tkac I, Starcuk Z, Choi IY, Gruetter R: **In vivo 1H NMR spectroscopy of rat brain at 1 ms echo time.** *Magn Reson Med* 1999, **41:**649-656.

13. Vanhamme L, van den Boogaart A, Van Huffel S: **Improved method for accurate and efficient quantification of MRS data with use of prior knowledge.** *J Magn Reson* 1997, **129:**35-43.

**Supplementary Results**

**
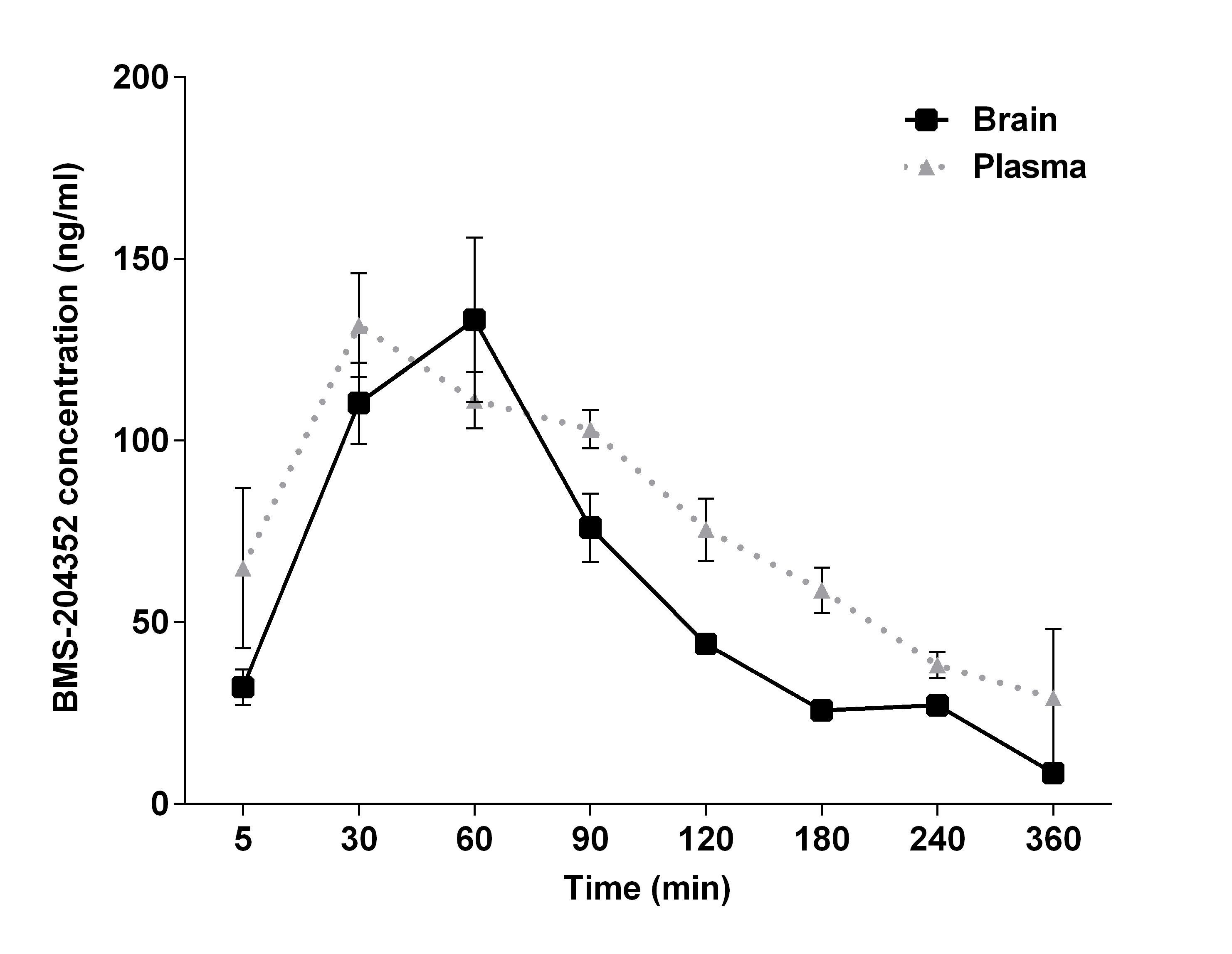
**

**Figure S1. BMS-204352 2mg/kg pharmacokinetic results.** Following intraperitoneal administration at 2mg/kg, plasma concentration *versus* time curve increased to an observed Cmax 139 ± 27 ng.mL^-1^ with a Tmax 30 min. A slightly delayed concentration time-course were observed in the brain (Tmax = 42 ± 16 min) with a Cmax 142 ± 43 ng.mL^-1^ but with an earlier decrease than in plasma. Tmax and Cmax were not statistically different between plasma and brain (p < 0.05). Mean BMS-204352 concentrations ± s.e.m. in plasma (**▲**, dashed line) and in brain (■, solid line) *versus* time in mice after an intraperitoneal bolus of 2mg/kg of BMS-204352.

**
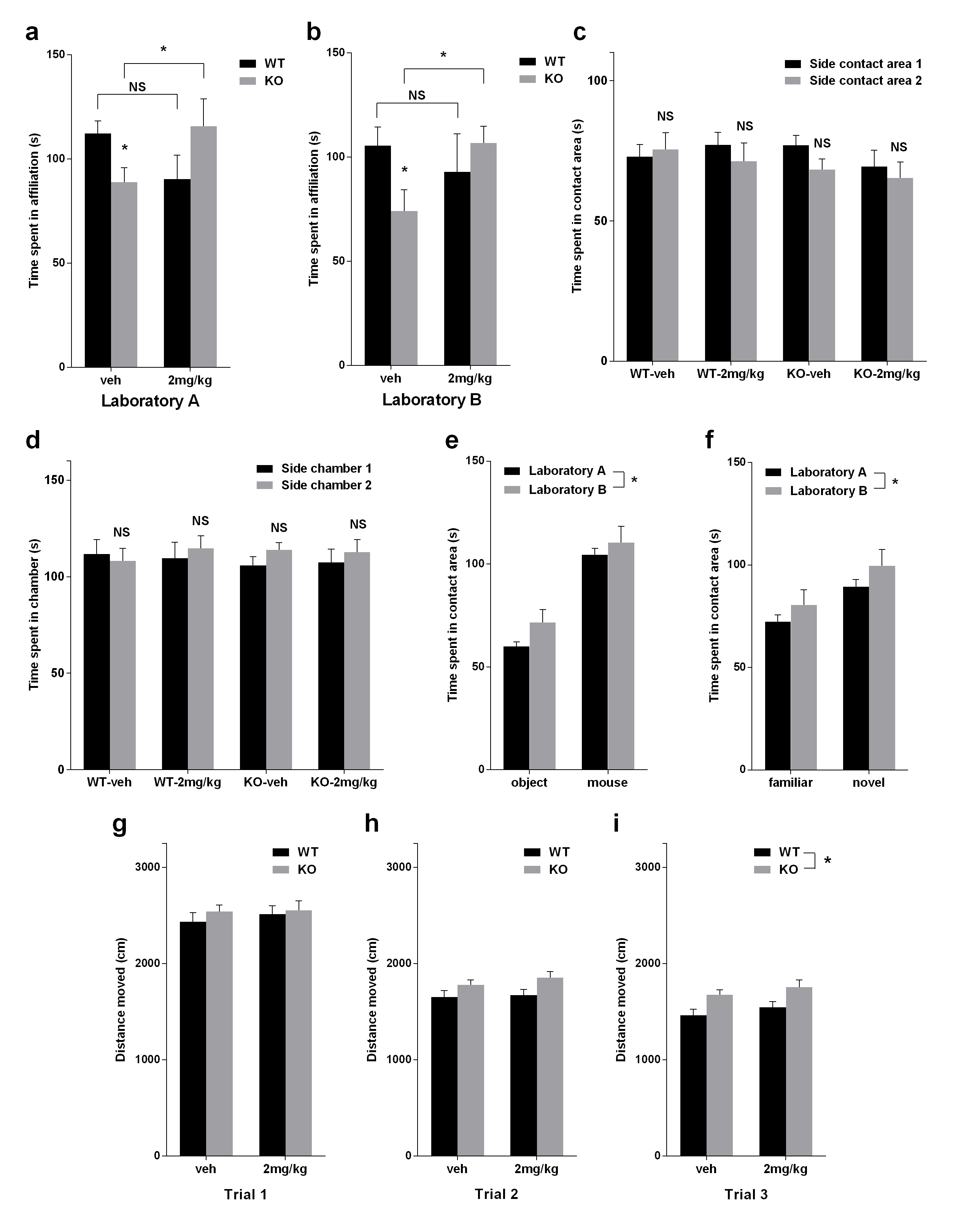
**

**Figure S2. BMS-204352 effects on Social behaviors.** Mice (3-5 months) were administered with BMS-204352 2mg/kg or vehicle (veh) and subjected to social behavioral tests 30 min after the injection. (**a-b**) Time spent in affiliative behaviors (sum of nose, body, and anogenital sniffing and allogrooming) during the direct social interaction test from Laboratory A and Laboratory B. Vehicle-treated *Fmr1* KO mice spent less time in affiliative behavior compared to WT, but BMS-204352 injection restored a normal social investigation. (*n*: WT-veh = 20; WT-2mg/kg = 10; KO-veh = 17; KO-2mg/kg = 10 from Laboratory A; WT-veh = 7; WT-2mg/kg = 6; KO-veh = 8; KO-2mg/kg = 7 from Laboratory B). (**c**) Preference for a side in trial 1 (habituation) during the three-chamber test was measured by the time spent in the compartments and (**d**) the contact areas. (**e-f**) MANOVA indicated that all experimental groups did not show a preference for any chamber in both laboratories. (**g-h-i**) Distance moved along trials. Main effect of laboratories observed during trial 2 and trial 3 without interaction between genotype, treatments and contact areas. NS, not significant; *p < 0.05 (*n*: WT-veh = 28; WT-2mg/kg = 13; KO-veh = 34; KO-2mg/kg = 17 from Laboratory A; WT-veh = 6; WT-2mg/kg = 6; KO-veh = 8; KO-2mg/kg = 8 from Laboratory B). Data represent mean ± s.e.m.

**
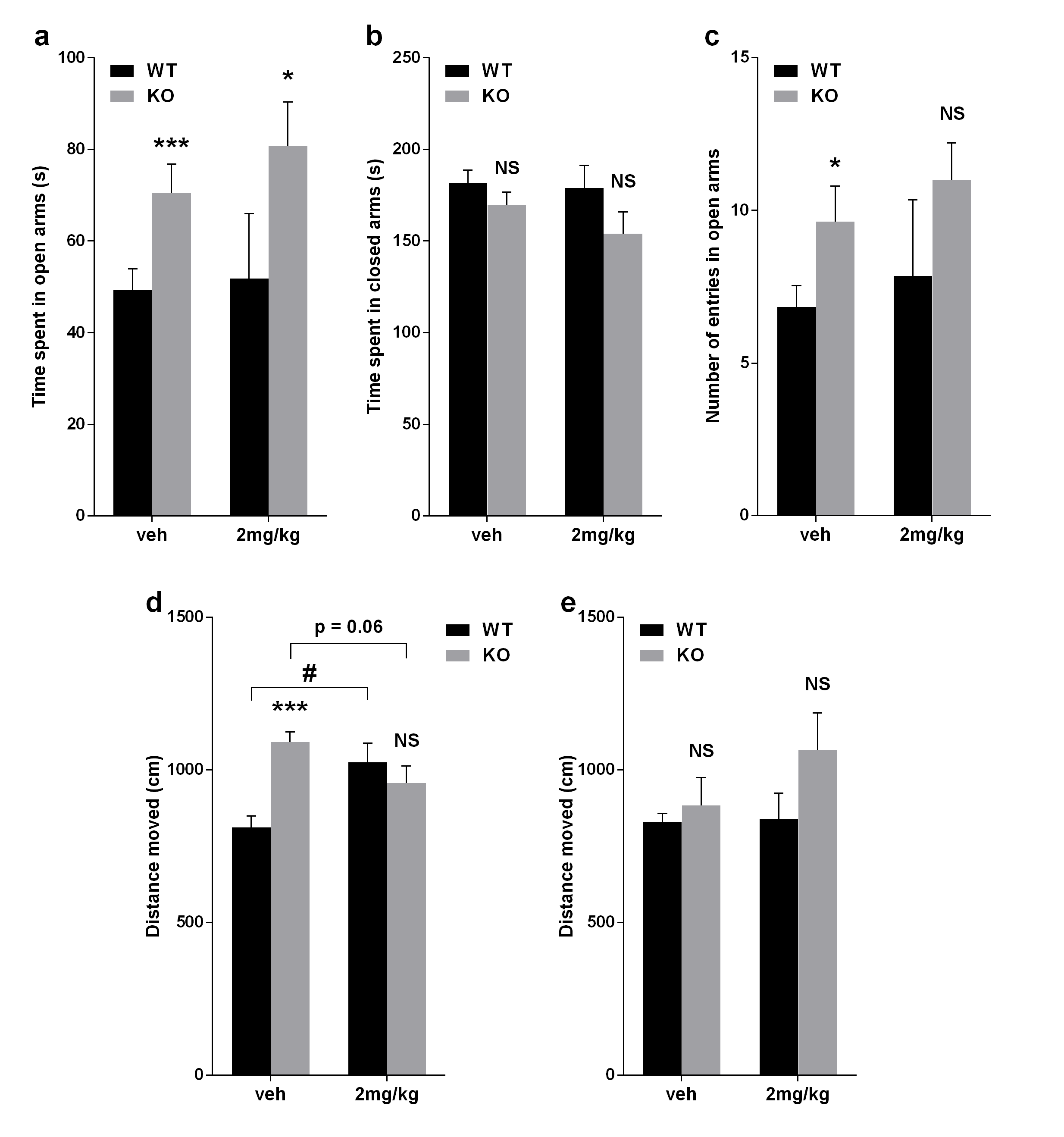
**

**Figure S3. BMS-204352 effects on Anxiety in the elevated plus maze and locomotion during the spatial memory Y maze test.** Time spent in (**a**) open arms and (**b**) closed arms was counted when mice were subjected to the elevated plus maze, (**c**) number of entries in open arms and (**d**) the distance moved. Significance was determined using two-way ANOVA with *post-hoc* PLSD test. NS, not significant; *p < 0.05, ***p < 0.001 and ^#^p < 0.05 (*n*: WT-veh = 32; WT-2mg/kg = 7; KO-veh = 32; KO-2mg/kg = 10). (**e**) Distance moved during the 5min-Y maze test. Significance was determined using two-way ANOVA with *post-hoc* PLSD test. NS, not significant (*n*: WT-veh = 7; WT-2mg/kg = 6; KO-veh = 7; KO-2mg/kg = 7). Data represent mean ± s.e.m.


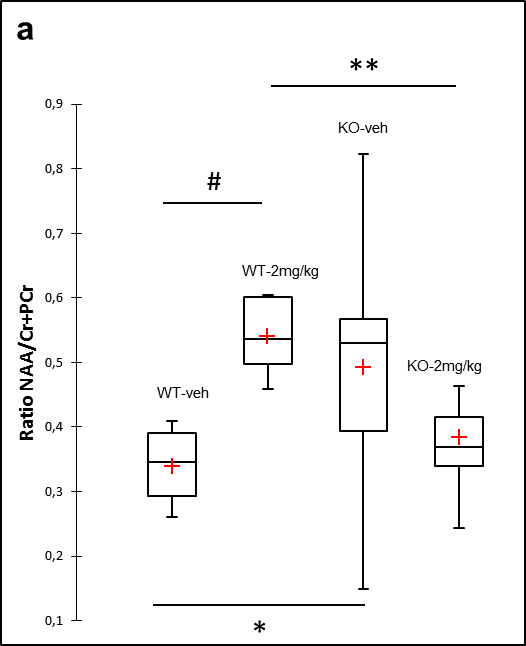

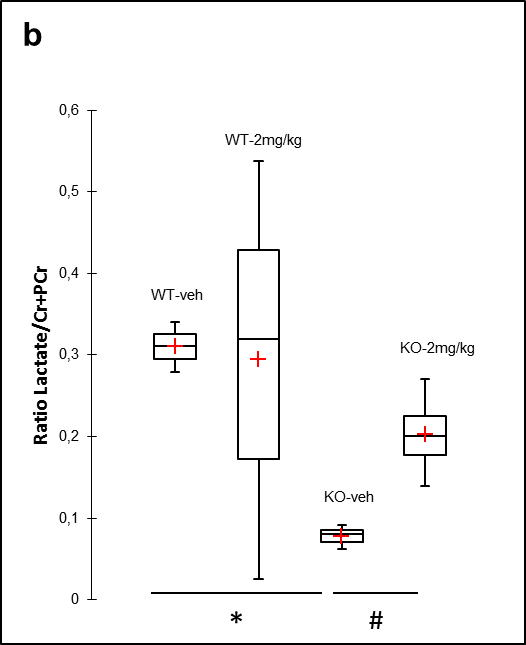

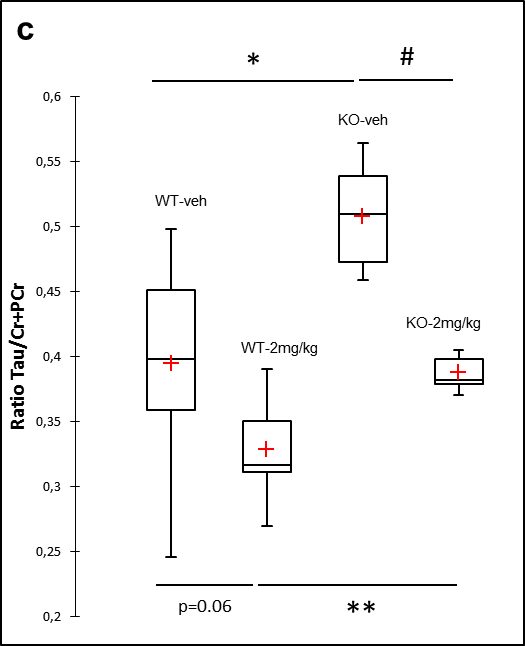


**Figure S4. BMS-204352 effects on N-Acetyl-Aspartate, lactate and taurine brain concentration.** Mice (3-5 months) were administered with BMS-204352 at 2 mg/kg or vehicle (veh) and subjected to MRS test after 30 min from the injection. Box plots (shown as the minimum, 1st quartile, mean, 2nd quartile and maximum values) represent metabolite concentration in arbitrary unit (AU) (**a**) N-Acetyl Aspartate, (**b**) lactate and (**c**) taurine, in vehicle-treated WT and KO as in BMS-204352 treated WT and KO. Significance between WT and KO was determined by pairs using Mann-Whitney test and impact of treatment was determined by Wilcoxon test. NS, not significant; *p < 0.05, **p < 0.01, compared to WT; ^#^p < 0.05 compared to treatment.


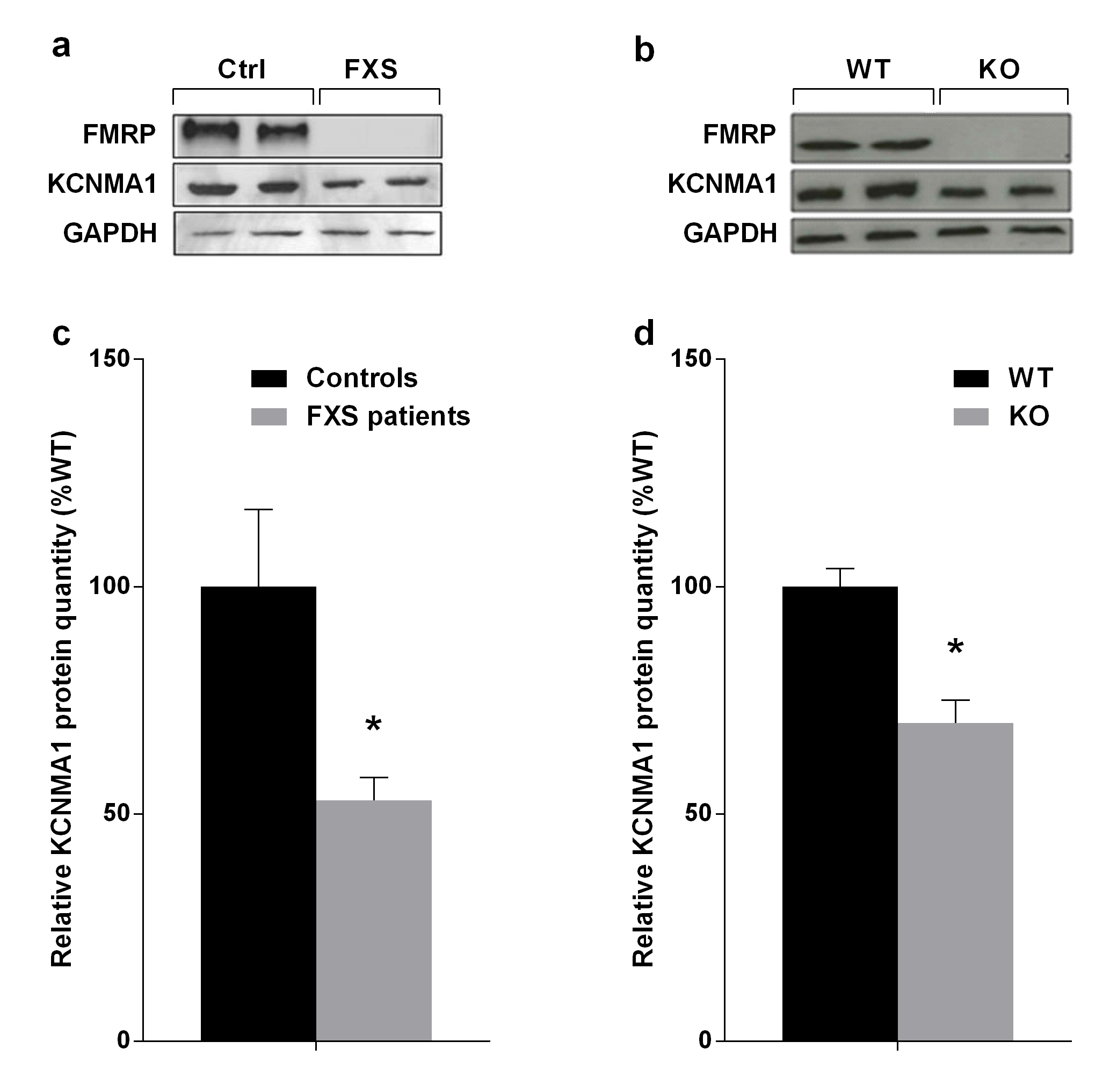


**Figure S5. KCNMA1 protein decreased in human FXS patients and in *Fmr1* KO mice.** Protein quantity was measured by western-blot (normalized by GAPDH housekeeping protein) in (**a**) human lymphoblastoid cells (Controls and FXS patients) as in (**b**) primary neurons culture of mice (WT and *Fmr1* KO). (**C-D**) In human FXS patients as in primary neurons culture of *Fmr1* KO mice, KCNMA1 is significantly decreased by ~ 40%. Significance was determined using one-way ANOVA with post-hoc PLSD test. **P*<.05; n = 10 mice per groups and n = 5 human individuals per groups. Error bars represent s.e.m.


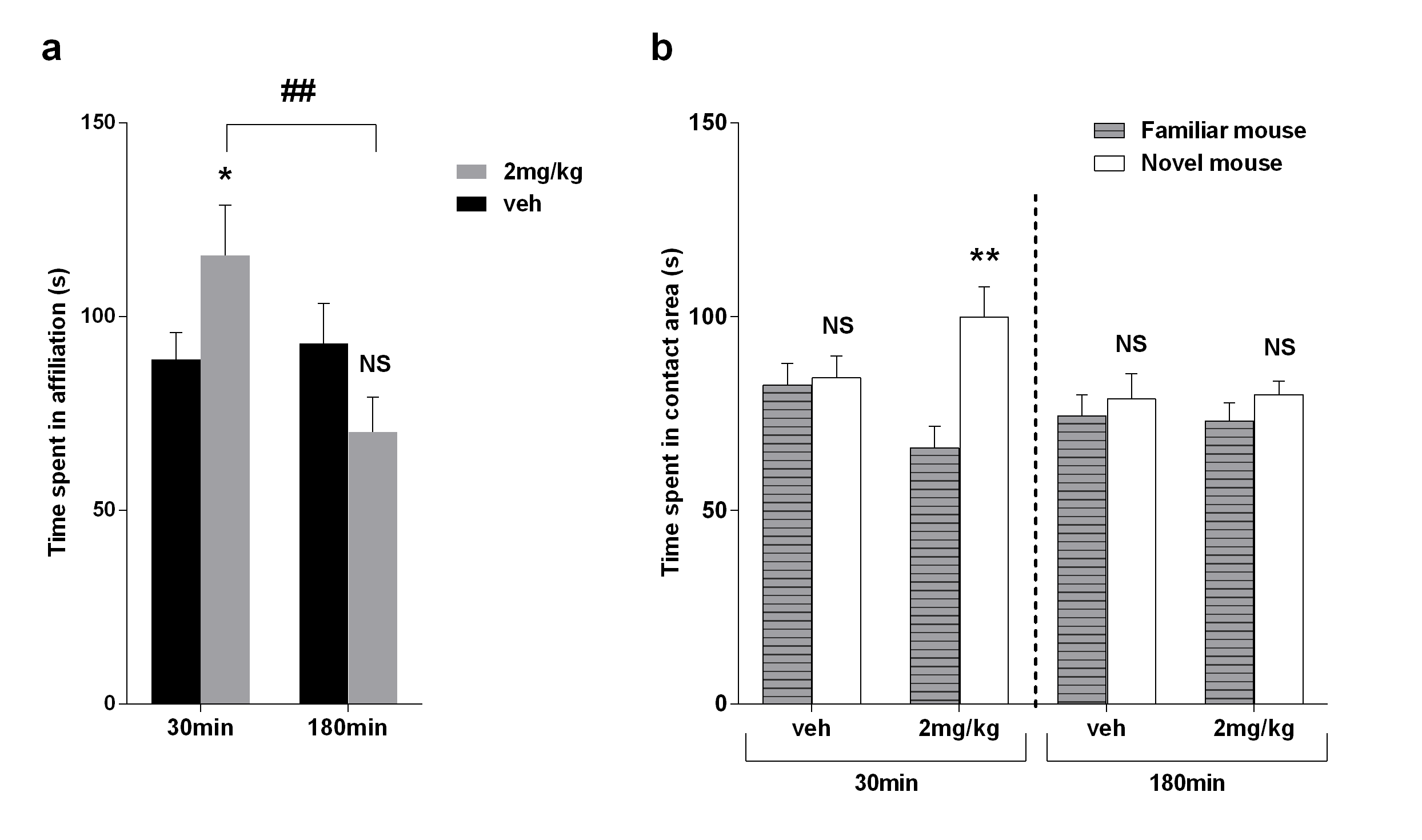


**Figure S6. Long time BMS-204352 effects on Social behaviors. *Fmr1* KO** mice (3-5 months) were administered with BMS-204352 2mg/kg or vehicle (veh) and subjected to social behavioral tests 30 min or 180 min after the injection. (**a**) Time spent in affiliative behaviors (sum of nose, body, and anogenital sniffing and allogrooming) during the direct social interaction test. BMS-204352 at 30min after injection restored a normal social investigation but not after 180 min. NS, not significant; *p < 0.05, ^##^p < 0.01. (**b**) Preference for a novel versus a familiar mouse in trial 3 (social novelty) during the three-chamber test, was measured by the time spent in the contact area when a stranger and a familiar mouse were accessible. Vehicle-treated *Fmr1* KO mice didn’t present a preference for the novel mouse. This preference was recue by BMS (2mg/kg) at 30 min delay after injection but not at 180 min. NS, not significant; **p < 0.01 (familiar versus novel mouse). (*n*: 30min : KO-veh = 34; KO-2mg/kg = 17 ; 180min : KO-veh = 10; KO-2mg/kg = 10). Data represent mean ± s.e.m.
